# Multi‐ancestry Genome‐wide Gene‐Vascular Risk Factors Interaction Analyses in Alzheimer’s Disease

**DOI:** 10.1002/alz.093041

**Published:** 2025-01-03

**Authors:** Annie J. Lee, Zuoqiao Cui, Zhengwei Song, Dolly Reyes‐Dumeyer, Philip L. De Jager, David A. Bennett, Julie A. Schneider, Vilas Menon, Yanling Wang, Rafael A. Lantigua, Martin Medrano, Diones Rivera Mejia, Ivonne Z. Jiménez‐Velázquez, Walter A. Kukull, Sarah A Biber, Adam M. Brickman, Giuseppe Tosto, Caghan Kizil, Lindsay A. Farrer, Jesse Mez, Jaeyoon Chung, Badri N. Vardarajan, Richard Mayeux

**Affiliations:** ^1^ Department of Neurology, Columbia University Medical Center, New York, NY USA; ^2^ Taub Institute for Research on Alzheimer’s Disease and The Aging Brain, Columbia University Medical Center, New York, NY USA; ^3^ The Gertrude H. Sergievsky Center, College of Physicians and Surgeons, Columbia University, New York, NY USA; ^4^ Department of Biostatistics, Mailman School of Public Health, Columbia University, New York, NY USA; ^5^ G. H. Sergievsky Center, Vagelos College of Physicians and Surgeons, Columbia University, New York, NY USA; ^6^ Department of Neurology, Vagelos College of Physicians and Surgeons, Columbia University, and the New York Presbyterian Hospital, New York, NY USA; ^7^ The Taub Institute for Research on Alzheimer’s Disease and the Aging Brain, Vagelos College of Physicians & Surgeons, Columbia University, New York, NY USA; ^8^ Department of Neurology, Center for Translational and Computational Neuroimmunology, Columbia University Medical Center, New York, NY USA; ^9^ Rush Alzheimer’s Disease Center, Rush University Medical Center, Chicago, IL USA; ^10^ Rush Alzheimer's Disease Center, Rush University Medical Center, Chicago, IL USA; ^11^ Center for Translational & Computational Neuroimmunology, Department of Neurology, Columbia University Irving Medical Center, New York, NY USA; ^12^ Department of Medicine, Vagelos College of Physicians & Surgeons, Columbia University, New York, NY USA; ^13^ School of Medicine, Pontificia Universidad Catolica Madre y Maestra, Santiago Dominican Republic; ^14^ Universidad Pedro Henríquez Urena, Santo Domingo Dominican Republic; ^15^ Universidad de Puerto Rico, San Juan Puerto Rico; ^16^ Department of Epidemiology, School of Public Health, University of Washington, Seattle, WA USA; ^17^ National Alzheimer’s Coordinating Center, University of Washington, Seattle, WA USA; ^18^ G.H. Sergievsky Center, Vagelos College of Physicians and Surgeons, Columbia University, New York, NY USA; ^19^ Department of Neurology, College of Physicians and Surgeons, Columbia University, New York, NY USA; ^20^ Department of Medicine (Biomedical Genetics), Boston University Chobanian & Avedisian School of Medicine, Boston, MA USA; ^21^ Framingham Heart Study, Boston University Chobanian & Avedisian School of Medicine, Boston, MA USA; ^22^ Takeda, Cambridge, MA USA; ^23^ Department of Neurology, The Taub Institute for Research on Alzheimer’s Disease and The Aging Brain, Columbia University Medical Center and The New York Presbyterian Hospital, New York, NY USA; ^24^ Departments of Neurology, Psychiatry, and Epidemiology, Gertrude H. Sergievsky Center, The Taub Institute for Research on Alzheimer’s Disease and the Aging Brain, Vagelos College of Physicians and Surgeons, Columbia University, New York, NY USA

## Abstract

**Background:**

Cardio and cerebrovascular risk factors (CVRFs) increase the risk of cerebrovascular disease and clinical Alzheimer’s Disease (AD), and over 70% of the patients with AD coincident cerebrovascular pathology. We previously found that *FMNL2* interacts with a burden score of hypertension, diabetes, heart disease, and body mass index (BMI) by altering the normal astroglial‐vascular mechanisms that underly amyloid clearance. Stroke, defined by history of a clinical stroke or brain imaging, is a moderately robust risk factor for AD and dementia. The goal here was to identify genes that interact with CVRFs, incorporating stroke as an additional factor, on AD in multi‐ethnic cohorts.

**Method:**

We conducted a genome‐wide gene‐CVRF score interaction analysis for AD, in 7,939 AD patients and 9,631 controls from eight multi‐ethnic cohorts of non‐Hispanic Whites, African Americans, and Hispanics including ADNI, NACC, NOMAS, WHICAP, EFIGA, and ROSMAP. A CVRF score was created from the first principal component of history of clinical stroke, hypertension, diabetes, and heart disease, and measured BMI. Gene‐based interaction test was performed with the adaptive gene‐environment interaction test. Results were summarized using a meta‐analysis. We investigated the association of pathological AD, amyloid‐β, or brain infarcts with gene expression and protein expression from the frontal cortex in ROSMAP using a generalized linear model. Age, sex, and the first three principal components were adjusted in the models.

**Result:**

The interaction of CVRF score with *FMNL2* on AD (*p* = 1.02E‐05) was identified and additional genes were identified to interact with CVRF score, including *SLC22A14* (*p* = 1.44E‐06), *AMMECR1L* (*p* = 2.74E‐06), *PRG3* (*p* = 2.76E‐06), *CFAP99* (*p* = 5.22E‐06)*, ADPGK‐AS1* (*p* = 8.58E‐06) and *BRINP1* (*p* = 6.29E‐06). *ADPGK‐AS1* and *FMNL2* gene expressions were associated with pathological AD (p = 0.004 and p = 0.0002). *FMNL2* and *BRINP1* gene expressions were higher in the brains of patients with brain infarcts (p = 0.025 and p = 0.006). *BRINP1* protein expression was associated with pathological AD (p = 0.0002) and was higher in the brains of patients with brain infarcts (p = 0.022).

**Conclusion:**

We identified novel candidate genes that interact with CVRFs on AD in multi‐ethnic cohorts. Understanding the interplay between genes, CVRFs, and AD has the potential to reveal novel molecular targets for prevention and treatment for AD.

